# Insanity

**Published:** 1845-01

**Authors:** 


I. Traité de Pathologie Cerebrale. Par *Scipion Pinel.* Paris 1844. 8vo. pp. 560.

A Treatise upon Cerebral Pathology. By *Scipio Pinel.*

II. First, Second, Third, Fourth, and Fifth Report of the Resident Physician of the County of Middlesex Pauper Lunatic Asylum at Hanwell, 1839-43.

III. Reports of Sir Alexander Morison, Visiting Physician of the Surrey Lunatic Asylum. 1842-43.

IV. General Report of the Royal Hospitals of Bridewell and Bethlem. 1844.

V. Report of the Metropolitan Commissioners in Lunacy to the Lord Chancellor. 1844.

In resuming the analysis of M. Pinel's new work, commenced in our last number, we have combined with it some consideration of the various "Reports," the titles of which are mentioned above, as tending to illustrate some of the subjects treated of in the latter part of the volume. Moreover, they are very important documents in themselves, as exhibiting not only what can be, and what has been done for the improved management of the insane, but also how much yet remains to do, before this can be considered as placed upon a satisfactory footing. It is understood that the Minister is about to introduce, during the ensuing Session, some comprehensive measure for the provision, regulation, and inspection of the various classes of establishments for the reception of lunatics: and, judging from the Report of the Metropolitan Commissioners, founded upon evidence derived from their recent visitation tour, not before it is needed. There are, it seems, 20,893 lunatics under confinement in England and Wales, of whom 16,821 are in the condition of paupers. Of this latter number 7,482 are alone provided for in receptacles especially devoted to the insane, the remaining 9,339, remaining in the workhouses, under, for the most part, every possible disadvantage. Even the magnificent establishment at Hanwell, and notwithstanding its dormitories are inconveniently crowded, is unable to supply the wants of the county in which it is situate; for while, in 1843, beds were made up for 1000 patients, the number of lunatics to be provided for amounted to 1429. Altogether there are 166 Lunatic Asylums in England and Wales, of which *seventeen only* are county asylums, 11 are supported in part by subscription, 1 is a military and 1 a naval hospital, and 136 are private asylums, licensed by the provincial magistrates or the Commissioners of Lunacy, forty-four of these latter establishments receiving paupers as well as private patients. Add to all this, the very considerable number of persons who are residing in private apartments under the *surveillance* of a keeper, and submitted to no system of inspection whatever, and of whose numbers therefore no return can be made, and we observe that there is indeed an immense mass of helpless misery to be legislated for. The Report not only demonstrates the lack of accommodation which exists, but the urgent necessity of a more rigid system of inspection. Licensed Asylums, whose management the magistrates have pretended to superintend, have been found by the Commissioners, in many instances, to be most disgracefully conducted: the patients not only being surrounded by the most disgusting filth, but subjected to all the cruelties of the ultra-coercive system, and that at a period when we had fondly imagined the employment of the fetter, the leg-chain, the restraint-chair, and the lash was for ever exploded. How slow is the progress of human improvement!

It will be recollected that M. Pinel arranges the affections of the brain into four classes, each distinguished by the derangement of some principal function of the organ. Thus there are *Lesions of the Understanding;* of the *External Senses and General Sensibility;* of the *Propensities and Instincts;* and of *Voluntary Motion.* The first we have already considered, and proceed to pass the others in brief review.

### CHAP. V.—LESIONS OR PERVERSIONS OF THE INSTINCTS AND PROPENSITIES.

The love of life is the strongest of the instincts implanted within our organization; and it is upon it, and upon all tending to encourage its development, that our laws and social institutions are based. But this instinct has its diseased manifestations, just as the understanding has its maladies. All the chimerical fears of hypochondriasm may be produced when its diseased condition is but slight, while, when the perversion becomes complete, we have suicide, a desire for self-destruction, just as imperious as is the instinct of self-preservation in a normal condition of the cerebral functions. Or, again, a desire to kill others becomes another symptom of the disease, a homicidal mania actuated by no motive whatever.

1. *Hypochondriasis.*—The author suggests that as this and similar affections are characterised by exaggerated fears concerning health and life, *biophobia* would be a more appropriate appellation; and we may thus speak of slight or severe biophobia instead of hypochondriasis, suicidal biophobia, homicidal biophobia, &c. Certainly a worse term than hypochondriasis can hardly be chosen. Of all the causes of this distressing disease M. Pinel considers an hereditary predisposition as the most prominent, "for if we go back to a period prior to the invasion of the disease, we find these patients, or their relatives, have been capricious, coleric, dull, or merry, without apparent reason, very liable to a variety of nervous ailments, and that there have been members of the family afflicted with insanity or epilepsy." He regards the disease as essentially an affection of the central portions of the brain, and that the serious accompanying affections of the thoracic and abdominal viscera are but the results of an aggravated degree of this cerebral lesion.

2. *Suicide.*—This species of suicide, resulting from a perversion of the instinct of self-preservation, is to be distinguished from suicide occurring in the insane, consequently upon illusions. Suicide is sometimes, as in the example of puerperal women, an acute affection, the desire of destruction continuing for some days, and then, if its fulfilment has been prevented by active surveillance, passing off and the patient becoming cured. At other times the act has been long premeditated, the instinctive love of life struggling against the effects of the lesion. The torment of an incurable disease, the long wished for leisure after an active life, the state of mind resulting from an exhaustion and abuse of sensual pleasures, or from the operation of various physical and moral influences, may one and all lead to this imperious desire of self-destruction. Occasionally we see persons surrounded by every comfort and means of happiness, place their affairs in order, take leave of their friends, and destroy themselves—some unperceived circumstance having in them given rise to this perversion of the instinct. Mutual suicide, generally occurring in the case of persons of opposite sex, influenced by love or extreme distress, has become very prevalent in France, to which the injudicious publicity given to these events in the newspapers has doubtlessly contributed. No age seems exempt; for suicide is committed from the age of 12 to that of 80, and the wells at the Bicetre have been closed in consequence of the increasing propensity among the aged persons for throwing themselves into them. The contagious effect of examples of suicide is indeed well known, and at some periods the affection has become epidemic.

The act of suicide is usually preceded for a considerable space of time by some of the various symptoms of hypochondriasis; but after a while the desire of death as a means of escape from real or imaginary misery becomes the dominant idea, and is dwelt upon with pleasure. But months or years of irresolution are sometimes passed in a constant struggle with this propensity, until the physical frame and mental power of resistance are worn out. In the acute form, no matter what the means of death, it is sought with avidity, and sometimes with incredible cunning; but, in the chronic form, some particular mode of destruction is determined upon, and none but it, as well as a certain time, place, &c. will suffice. Perhaps about one-half of those persons who attempt suicide do not succeed; and the revulsion engendered by the panic they are then seized with, often operates as a, at least, temporary, and sometimes permanent, means of cure.

The author believes modern civilization to be a great encourager of suicide, by reason of the forbearance which it manifests towards those who attempt it, and the notoriety it obtains for them in the public prints. He contrasts with this the rigid laws of the Greeks, Romans, and St. Louis.

The disposition to suicide existing in many insane persons has always proved a source of much inquietude to their friends and attendants; and the adoption of the most rigid restraint was long considered as the only safeguard. It will be seen that prevention by this means must be rarely practicable, for the very instruments of restraint have been often converted into means of destruction; and it is a most happy circumstance, that in proportion as coercion of this description has been disused, the proportionate number of cases of suicide has diminished, and the number of cures augmented. This is the concurrent testimony of all modern insane establishments. The Bethlem Report states :—

"The experience of the last year adds another confirmation to the now generally received opinion, that mechanical restraint is an exciting cause of suicidal propensities; and though it may for a time restrain the attempt, it fosters and strengthens the desire it is intended to control. During the last year so large a number as 81 patients, or more than 28 per cent. of those admitted, were reported as having suicidal tendencies; and 37, or 13 per cent. had actually attempted suicide prior to admission; and it is highly creditable to the officers of the hospital, and an indisputable proof of the vigilance of the attendants, that no attempt at suicide has occurred during the year. The last case of suicide was that of a female in 1840: no case amongst the males has occurred since 1822. No stronger evidence can be given of the tendency of mechanical restraint to excite suicidal attempts than that supplied from the records of the hospital; from which it appears that during 20 years, from 1750 to 1770, when every patient was under restraint, the suicides were in the proportion of 1 in 202, whereas, during the last 20 years, the proportion has only been 1 in 963." 53.

Dr. Conolly's Second Report from Hanwell contains some valuable observations upon this head.

"Nine suicidal cases are among the admissions of the past year. It affords gratification to the physician to be enabled to state, that in all of these cases means have been found to soothe and comfort the minds of the patients, and, apparently, to reconcile them to life. Their restraints have in all cases been immediately removed, and in no case resorted to again. They have been watched, so long as it was deemed necessary, during the day, placed in rooms with other patients by night, and frequently visited. Every instrument of danger, or obvious means of self-destruction, has been kept out of their way; and no measure likely to restore cheerfulness has been omitted. This is the general plan resorted to. But in almost every case of this kind the bodily health is manifestly disordered; and when proper remedial means are applied, the propensity to suicide is weakened, or disappears. Redness of the tongue; disinclination for food: irritable bowels: feebleness and emaciation: cold hands and feet; are not uncommon symptoms. In other cases, a loaded tongue, obstinate constipation, and appearances of hepatic disorder, are observed. Both of these descriptions are chiefly applicable to patients between 40 and 50 years of age. When submitted to proper remedial treatment, they commonly improve, although very slowly; the health being usually, or at least often, impaired beyond the hopes of perfect restoration. It is impossible for attention to these patients to be too vigilant, but not at all impracticable to establish such systematic vigilance on the part of the officers and attendants as will afford security. To torment these unhappy patients with bodily restraints, would only fix the morbid determination more deeply in their minds. Disposition to suicide is not uncommon in women at a much earlier age, and is usually associated with some uterine irregularity, to which may be added moral causes of various kinds." 40.

3. *Homicide.*—This is a perversion of the affective faculties, which is often accompanied by a complete integrity of the intellectual powers. It frequently precedes suicide; and, where this is not the case, the homicide often voluntarily delivers himself into the hands of the law. These cases of motiveless homicide are the most frequent, and to be distinguished from those in which the intellectual powers are perverted, and murder is perpetrated under some delusion. Esquirol, Gall, and other authors, cite abundant examples of each of these forms. This distinction, however demonstrable to the scientific enquirer, has been received with difficulty in courts of justice; and is obviously open in its practical working to abuses. Nevertheless, no fact is better established now than that this impulse to kill may exist independently of delusion, and yet uncontrolable by its unhappy subject, who has been often known to seek the assistance of others to save himself or them when he has found the paroxysm approaching. These persons, if properly watched, and separated from sources of irritation, may frequently become cured of these terrible propensities.

Dr. Pinel dwells upon the importance of an improved study of this disease in reference to legal medicine, and records some of the confused and contradictory conclusions the courts of law have arrived at, parallel instances of which could easily be supplied from this country also. Much practical difficulty attends the case.

"It is certain that such monstrous actions must at first carry terror into the minds of the administrators of justice, who fear lest every criminal may shield himself under the impunity of these examples. For, often in homicidal mania, as in crime, there is premeditation, hesitation, a consciousness of murder. It is only from the conjunction of circumstances which have preceded, accompanied, and followed the act of homicide, that evidence and conviction of non-culpability, and of the true character of the mania, can be obtained. The physiology of the brain is as yet too recent. Too short a time has elapsed since it has taught us that the affective, like the intellectual faculties, are but the expression of its normal condition, and may become solely the subjects of perversion, just as certain functions of the understanding may—for this knowledge to have penetrated among the masses and become vulgar: but it will do so." 322.

4. As this perversion of the affective faculties may occur as a grave disease in one possessed of his reasoning powers, so may it complicate the condition of those in whom these too have become perverted. Thus it is by no means uncommon in the insane; and it has been observed that those who were, prior to their insanity, of a mild demeanour and good moral conduct, form the majority of the subjects.

5. *Incendiary Mania.*—Marc pointed out this example of the destructive instinct in 1823, and numerous cases corroborative of the propriety of referring it as the above to the perversion of the affective faculties. The affection is frequently propagated by imitation, and often rages almost epidemically. At the commencement of insanity many patients are seized with the desire of destroying by fire; while, in other cases, individuals, possessed of reason, but of limited capacity, take great delight in committing the act. The following observation was confirmed during our recent trials for incendiarism in many instances.

"Individuals of a very limited capacity may be made the instruments of malefactors for the accomplishment of their guilty designs. It is for a trifling reward, a piece of bread, often as a mere joke, that these weak beings allow themselves to be persuaded. They may be termed thoughtless incendiaries. Others however merely obey the impulse of false ideas, insane conceptions, of which we have a remarkable example in the case of Jonathan Martin, who fired York Minster."

6. "*Manie de Caractère.*"—Manie raisonnante of Pinel, and Moral Insanity of Pritchard. The author thus characterizes the condition.

"I think we may term manie de caractère that slight perversion of the instincts and affections which renders the individual a scourge to all around him, and which is yet unattended with mental delusion. These are turbulent, unmanageable beings, coleric in disposition, committing various censurable acts, which they are always ready to justify by plausible reasons; and who become to their friends and family a continued source of inquietude and grief. They commit mischief for amusement, malice, or wickedness, and are incapable of application or labour. They break, disarrange, and destroy everything. . . . . Individuals afflicted by this partial perversion of the disposition, commit out-of-the-way actions, and maintain the most singular and absurd conversation, well knowing all the while what they do and what they say. The understanding suffers no lesion: the patient is enabled to justify his proceedings with a surprising connection and lucidity of ideas and expressions. There is but an instinctive perversion—a general exaltation of the bad propensities, but rarely to the extent of insanity."

Such persons often have violent outbreaks of short duration directed against a variety of subjects; and they have usually sufficient control over their violence, so as to escape from some of its consequences. There seems an innate perversion of the propensities, but not to an extent to warrant seclusion. When this perversion of the affections complicates ordinary insanity, the lunatic becomes the most insupportable of beings, creating eternal confusion and quarrels among the other inmates and their attendants, which indeed seems the chief object of their lives.

7. The propensity to *thieve*, so frequently exhibited in persons whose circumstances place them above the necessity, and many other perversions, all belong to the same category.

### CHAP. VI.—LESIONS OF VOLUNTARY MOTION.

These may exist in the condition of exaltation, of debility, and of intermittence. In the first, we have placed maniacal furor, tetanus, convulsions, and chorea; in the second, paralysis, delirium tremens, senile tremor: in the last, epilepsy and hysteria.

It is obvious we cannot pursue the author's examination of all these important diseases; and will content ourselves with an abstract of his opinions upon *general paralysis* or *paralytic cerebritis*, to which he attaches considerable importance on the score of their originality. The Bicêtre has afforded him ample opportunities of studying these cases, two wards being expressly devoted to their reception. He believes very different pathological conditions have been confounded under one vague term, and recognizes six distinct forms of the affection, differing in anatomical character, progress, and symptoms.

The *first* and rarest of these has not hitherto been described, and is that which is associated with acute inflammatory action in the periphery and central portions of the brain, and which resists all the resources of art. 2. In this we have the affection of the brain in a chronic condition, with consequent ramollissement, of the grey substance especially, the membranes and white portions of the brain being also affected. The atrophy which results is accompanied by a general sinking-in of the brain, leaving a space between its periphery and the skull-cap. M. Parchappe, after his numerous investigations, came to the conclusion, that general paralysis essentially arose from a ramollissement of the middle layer of the cortical substance of the cerebral hemispheres. 3. With all the symptoms of acute phlegmasia there is found after death a condition of hypertrophy of the white portion of the brain, its volume and density being both increased, and the augmentation proceeding from the base upwards, so that at last the convolutions become flattened by reason of pressure against the cranium. The grey substance becomes diminished, and no vascularity of the white exists. This change in structure, when chronic, has very frequently been observed by various authors in epilepsy. 4. This is exactly opposed to the last form, consisting as it does in atrophy of the white substance of the brain. The hemispheres are often reduced to one-third of their volume, leaving more than an inch interval between their surface and the interior of the cranium. In most cases there is phlegmasia of the grey substance; but, as in the case of hypertrophy, the white portion is that in which change in bulk is observed. There are often meningeal hæmorrhage and serous effusions present, arising probably from the obliterated condition of the vessels within the convolutions. 5. In this the paralysis is *intermittent*, returning only at intervals of six months or a year, and then again disappearing for the like, or a shorter period. 6. In this the disease is cured, either by influence of the treatment employed, or, much more probably, by reason of the favorable constitution of the patient. Such cures are rare indeed, but they are occasionally operated upon patients seemingly at the point of death.

These various forms are illustrated by means of twenty-four well-narrated cases; so that the chapter forms a valuable monograph upon the subject well worth consulting.

"We are now in a position to observe that in general paralysis, as in all the other diseases of the nervous system, the changes of structure are very variable, and that they present very different types, each having very opposite aspect and characters, although the prominent symptom in all is paralysis, first of the organ of speech, and afterwards of the sphincters and limbs. We may see that the identity of lesion is a chimera that we must renounce, and the identity of the seat of disease is hardly more tenable."

Dr. Pinel thus speaks afterwards concerning the *Seat* of the disease.

"We observe that it is especially the muscles of the tongue, larynx, œsophagus, and sphincters of the anus and bladder which are first paralysed; and that it is only some time after that, first the superior and then the inferior extremities become affected. We feel no doubt that the affection commences with a lesion of the olivary bodies, or some of the nervous bundles emerging from these, and afterwards propagates itself to the rest of the hemisphere; or if the affection originates in the periphery, it must involve some filaments which are in relation with the olivary body. One must believe, from their singular configuration, the kernel of grey substance forming their centre, and from their development in man alone, that the olivary bodies, whose function is as yet problematical, must preside over some faculty peculiar to man—the articulation of laryngeal sounds. The nerves of the pharynx, larynx, and tongue spring from them, and it is the lesions of these organs which proclaim the advent of general paralysis. The facial arises in the same point, and the trembling of the lips and cheeks is very remarkable among these paralytics. Bell regards the olivary bodies as destined for respiration because the pneumogastric obtains thence parts of its origin, and the lesion of this nerve explains the inertia of diaphragm, the slow respiration, languor, torpor of the heart, and paralysis of the sphincters. * * * * The changes in the olivary bodies are as well characterized as those of any other of the nervous centres; but their examination, perhaps from difficulty of appreciating these, is completely neglected in autopsies. Nevertheless, Guislain, Esquirol, and others, have remarked their induration in epilepsy. In general paralysis their appearance and volume are in exact accordance with those of the other portions of the brain. In atrophy hardly a vestige of grey centre is discernible, while in hypertrophy they are large and projecting. We doubt not that in the stammerers and dumb these parts would present well marked changes." 396.

### CHAP. VII.—LESIONS OF THE EXTERNAL SENSES AND OF GENERAL SENSIBILITY.

These, as the other lesions of the nervous system, may manifest themselves by a state of exaltation of the sensibility—the hyperesthesia of Andral; a state of depression—anesthesia; and a state of perversion—illusions, painful affections.

Examples of an excessive excitability of one or more of the external senses, and especially of the general sensibility, are of common occurrence in patients who have become exhausted by excessive study, watchings, inordinate passions, and may also arise from an entirely opposite class of causes, as indolence, the privation of a customary stimulus, as tobacco, &c. A too rigorous diet maintained during convalescence may determine the same phenomena, as in fact may whatever contributes to the production of a defective or vicious hæmatosis—the nervous system being liable to inordinate excitation just in proportion as it is deprived of its due supply of highly arterialized blood. Hence the irritating effect which prolonged and chronic diseases exert upon the system. Partial or general diminution of sensibility is likewise of frequent occurrence, depending upon some more or less considerable changes in the nerves supplying the parts or in the centres whence they emanate. In considering perversions of the external senses, we must distinguish *illusions* from *hallucinations*. In the production of the latter the brain is alone concerned; while, in the former, the sensibility of the extremities of the nervous system is perverted, and conveys false impressions. A man in a healthy state of mind may be the subject of an illusion of the senses, but his reason corrects the error. This is not the case with the lunatic, and illusions of the various senses are constantly complicating their cases. The only example M. Pinel presents of a perverted condition of the general sensibility is the horrible disease *hydrophobia,* but his description of it is meagre, and presents no novelties. Of perversions of general sensibility accompanied by pain, the various forms of neuralgia, hemicrania, clavis hystericus, rachialgia, &c. are examples. Some of these may become epidemic.

"There prevailed in Paris fifteen years ago an epidemic of local pains in the palms of the hands and soles of the feet, to which the name of acrodynia was given. The patients compared this pain to that produced by the insertion of pins or needles. The skin became red, and insensible, and peeled off in scales. This epidemic was replaced by the influenza and cholera, and has not re-appeared since."

### Chap. VIII.—Of the Causes of Cerebral Diseases.

Dr. Pinel distinguishes these into predisposing, moral or functional, and physical causes: the last comprehending all the changes occurring in the brain, and the re-actions of the various viscera upon that organ.

### *Predisposing Causes.*

*Hereditariness.*—The author observes that the brain is one of the organs in which hereditary predisposition exhibits its most fatal influence, and that this is much more marked among the higher than among the lower classes. The highest families of France, obliged by their position in society to confine their alliances within a narow sphere, have become extinct; or, where this is not the case, the intellectual powers of their representatives have much degenerated. The tables conjoined to the Reports before us attribute the occurrence of Insanity to hereditary predisposition in a much less proportionate number of cases than it is usually believed to exist in; but it is often very difficult to ascertain how far this is the case in the poor and ignorant classes to which they relate. Amid the large assemblage of insane at the Salpêtrière, it is not uncommon to see two sisters, the mother and daugter, and sometimes the grandmother. Many members of the same family become mad under the same circumstances, or at the same age. If we could obtain exacter statements, we should doubtless find, that not only insanity, but all the acute and chronic affections of the brain, as apoplexy, epilepsy, &c. are often to be traced to hereditary origin. Great as is the influence of this predisposition, it would be very rash to suppose that children born of parents who have been insane must necessarily become so, especially if only one parent has so suffered. Education and the avoidance of the exciting causes which have proved to be mischievous, may do much in modifying the predisposition. Nevertheless, often it is so strong, that no care will avert the occurrence of the disease, even under the most favorable circumstances.

2. Various other causes predispose, as the *puerperal state* especially. It is not uncommon to see women in the Salpêtrière who have become insane after five or six confinements. The *critical age* is a powerful predisposer to cerebral affections.

"There are also certain dispositions of the mind and character to which the ready development of many cerebral affections must be attributed. Such are the natural infirmities of a weak intellect subjected to the domination of evil propensities, impetuous passions, a direction of the attention to a limited number of speculative religious or political ideas, the anxieties attendant upon most extensive enterprizes, the events which fix public attention, and which influence or change the popular ideas. It would not be difficult to retrace, among the admissions into our various establishments, from the period of the Revolution of 1789 to the present time, the form of insanity especially appropriate to the exaltation of ideas prevalent at each epoch." 450.

3. An anormal predominance of certain parts of the brain may give rise to a perversion or exaltation of certain of its propensities, and thus lead to some form of insanity. In acknowledging, in spite of many exceptions, as a general rule, that marked predominance of various regions of the brain is accompanied by corresponding manifestation of certain faculties and propensities, M. Pinel objects to the localization of Gall as too minute, while the relations of the bony vault with the convolutions are too remote and too variable for any practical purposes.

*Moral or Functional Causes.*

Causes operating by the exercise of the brain's own functions are those which produce its most serious maladies. All writers upon insanity place the operation of moral causes in the very first rank; and thus it is not in the extremes of life, but at those periods when the intellect and passions possess all their activity, that we find the most important diseases of the nervous system develop themselves. Moral causes are as various as the functions of the brain itself, of which, indeed, they are but exaggerations.

The great influence of moral causes explains in part the greater prevalence of insanity among women. Even in the Hanwell Asylum, where various circumstances may influence the parishes in sending male rather than female paupers, of the 2,588 admitted between 1831-43, 1279 were males and 1309 females; but in Bethlem, where un-restricted admission prevails, the females have predominated 47 per cent., and in St. Luke's 33½ per cent. Among the admissions, moral causes are also found to be much more proportionately prevalent in the one sex than in the other. This is a reason too for the greater curability of insanity among women.

"Among the various causes, some act instantly, producing at once delirium, convulsions, or paralysis, such as sudden terror, paroxysms of rage, sudden loss of wealth, &c.; while others produce their effects more imperceptibly and slowly. Grief, jealousy, religious creeds, political ideas, only produce an alteration in the cerebral functions by their long-repeated action. In insanity the delirium establishes itself thus gradually, becomes concentrated, and at last only explodes, when reason has lost all mastery over it." 456.

*Physical Causes.*

M. Pinel describes as the Physical Causes of the Diseases of the Brain, certain changes in its circulation or structure which in ordinary language are represented as the substantive diseases themselves. In fact, it matters little whether we describe, as he has done, first the aberrations of the various functions of the brain, and then refer back to the various changes in the anatomical conditions of the organ as the causes of such; or whether, as is usually done, we term the proximate cause the disease itself, and then describe the different functional derangements it gives rise to. He enumerates as physical causes, Congestion, Inflammation, Ramolissement, Induration, Cerebral Hæmorrhage, Cerebral Œdema, acute and chronic Hydrocephalus, and organic degenerations of the brain, as Tubercle and Cancer. All these are well described at length, but not in a manner to call for farther notice from us. We may, however, transcribe one or two of the author's general observations.

"In diseases of the brain, we may repeat with confidence, the pathological changes present general and determinate characters; and we must no longer look for identical lesions in identical diseases; for example, that there should be peculiar lesions in insanity, others in epilepsy, others in general paralysis, &c. To seek this is to pursue a chimera which facts do, and will more and more, destroy. In all cerebral affections, acute and chronic, the nervous pulp undergoes successive disorganizations (deformations) which may now be reduced into general laws. But an inevitable consequence of this leading fact, is a result we have many times indicated, and shall not fear to remark again whenever occasion offers; namely, that it is not according to the anatomical nature of the lesion, but accordingly as it involves this or that order of fibrous bundles or protuberances, of which the brain is composed, that you will observe the differences and speciality of these diseases. Accordingly as these fibres or prominences are intelligent, motory, or sensory, as they are more irritable in certain portions of the cerebro-spinal axis than in other portions, and their lesion is solitary or complex, you will have, in proportion to the duration and extent of the lesion, diseases differing much from each other, or complicated with symptoms in exact relation with the lesion, although the anatomical characters may be the same. The diseases of motility may thus have their *seat* wherever there are motory nervous fibres, and the diseases of sensibility wherever those of sensation exist.

"The acute or chronic symptoms correspond then to the acute or chronic lesions of the nervous pulp. When the alteration is but local or superficial, a very limited disorder of intelligence, locomotion, or sensibility is produced. When it is more extensive, and penetrates deeper into the cerebral mass, the disordered condition of the three functions is more pronounced: delirium complicates the lesions of motion and sensation which are expressed by permanent symptoms, as in paralytic cerebritis. When the sensitive and motory fibres, which are in so intimate connexion thoughout the mesocephalon, are simultaneously attacked, and the progress of the disease is more or less acute, you observe all the spasmodic and convulsive affections, and when the disease is more chronic or intermitting, the various epileptic and hysterical phenomena. In order that convulsions should be produced, there must be simultaneous lesion of the sensitive and motory nervous fibres."

Of *Ramolissement* occurring in the *aged*, M. Pinel observes:

"Ramolissement has been observed at every epoch of life; in the infant, as well as in the adult and the aged. It is tolerably frequent, especially towards autumn, in the infirmaries of the Salpêtrière, and there presents in its progress and its anatomical characters the effects and traces of the general debility of old age. It is rather a senile decomposition than an inflammatory process. This is the case with almost all the phlegmasiæ of the aged: for, in them, pneumonia is latent, without cough, pain, or expectoration, and erysipelas is indolent, requiring stimulation. So with cerebral ramolissement, it is asthenic, not having the power to become inflammatory."

### Chap. IX.—Treatment of Cerebral Affections.

This is naturally divided into three portions.

"1. The *therapeutical* treatment comprehends all descriptions of medication suited to combat the diseases of the brain, especially at their commencement, so as to prevent their passing into the state of incurability, and the access of the fatal complications which do not fail to follow. It seeks the best curative indications according to the number and gravity of the symptoms which present themselves. This treatment should be more active than it has hitherto been, since we find nearly all the diseases of the brain degenerate into an incurable state.

"2. The *functional or moral* treatment, which is the opposing certain ideas by other ideas or emotions, is applied with success to chronic or partial insanity, monomaniæ arising from superstition or vanity, &c.; in which the brain, having formerly suffered a slight lesion, continues incapable of reasoning by the force of habit or want of exercise. Such patients are often met with in large establishments for the insane, as well as among the higher classes of society.

"3. *Hygienic* treatment especially concerns those individuals whose malady compels a complete isolation—the insane, whose material condition we must render as favorable as circumstances will admit of. Hence the necessity of hygienic regulations for the arrangement of receptacles, and for the due classification of the inmates, the management of the diet, exercise, employments, and all other things which concern these patients." 494.

We need not quote M. Pinel's mode of treatment of the various other cerebral diseases, which has in it nothing peculiar; but will confine ourselves to his observations upon the treatment of the insane.

### TREATMENT OF INSANITY.

THERAPEUTICAL. *Bloodletting.*—Although this is required in acute or furious mania, it requires to be used with caution, as amelioration from its employment is often of short duration, and, if it be too long persisted in, the gravity of the case becomes augmented. In women whose catamenia are suppressed we should apply leeches to the thighs at their usual periods, when an exacerbation of symptoms always takes place. General bleeding, which may be required at the outset of the case, must afterwards be replaced by leeches or cupping, applied to the back of the neck, the thighs, or region of the heart. General bleeding is very seldom employed in Bethlem, or other English asylums.

*Tepid Baths,* used for prolonged periods, applying at the same time cold to the head, are attended with great advantage. *Cold* baths and the *douche* are of much more questionable utility, frequently giving rise to pulmonary or cerebral congestion. The douche has enjoyed an undeserved celebrity.

"If some success has been obtained from this procedure as a means of correction, I can unhesitatingly state that the effect is almost always the contrary to that which has been desired. At the Salpêtrière the employment of the douche is a constant cause of exacerbation. The patients become excited, vociferate and abuse those who administer it; or sometimes supporting it with a forced calmness, which is no less hurtful. I have observed women bear it better than men: at the Bicêtre it cannot be practised for even a short time without producing a state of congestion, or causing the patient to express the most terrible suffering; but, at the Salpêtrière, women are seen to bear it for more than a quarter of an hour without any emotion being caused, while others defy its effects and tire out the physician. As a curative indication it is a means whose consequences are dangerous, by reason of the intestinal and pulmonary congestions it produces. Better effects may be obtained from a narrow streamlet, or from water falling guttatim, the patient being placed in a comfortable manner in his bed. The application of ice to the head is an excellent means of calming irritation of the brain." 499.

Dr. Conolly states that the douche is now seldom resorted to at Hanwell, producing no good effect beyond that of the shower-bath, and distressing the patient much more. He finds the *shower-bath* the most effectual means of subduing violent excitement: the patient being placed up to the middle in warm water. Its application is however somewhat difficult, and it should seldom be employed as a punishment.

"It should be suspended when the patient appears overcome, and instantly renewed when symptoms of violence occur. A strong shower, continued even for a minute, has sometimes considerable effect; and it is never many minutes prolonged without careful observation of the patient's state. After four or five applications of this kind the patient becomes entirely subdued; and should then be taken out of the bath, rapidly dried, warmly covered up, and put into bed, with every possible demonstration of kind attention. Calmness and sleep are the usual results: and more permanent effects frequently follow. A bath of this kind seems to produce a moral as well as a physical impresssion; being succeeded, in recent cases, by tranquillity for a few days, and in chronic cases, by quietness and improved behaviour for many weeks, and even for months."—
—*2nd Report.*

*Purgatives.*—These have enjoyed a reputation in the treatment of mania for ages. Their use, during its acute stage, requires circumspection, as the intestinal mucous membrane often participates in the general excitability. When the nervous action seems blunted or suspended, we may proceed more boldly. Various mild aperient drinks are desirable.

*Emetics* have been much vaunted, but they have been often employed at the Bicêtre in large doses with little avail. They are most useful in small doses in cases of mild melancholia, slight hysteria, and attacks of nervousness in women.

*Counter-Irritation.*—In the acute stage exutories are of little avail: but they are of use at a later period, and especially in puerperal mania. Much has been said of the application of the actual cautery to the nucha or to the crown of the head. The author has frequently seen it employed by Esquirol, with little or no effect, unless a moral one, when it excited fear. Small derivatory effect results, as little or no suppuration follows the fall of the eschar.

Dr. Conolly speaks highly, in cases of excessive excitement, of shaving the head and applying the ung. ant. tart. freely to the scalp. All natural derivations are useful, as cutaneous eruptions, a loose condition of the bowels, the menses, hæmorrhoids, &c.: and when these are absent, in the chronic stage, large exutories producing abundant suppuration should be maintained.

In the case of *Illusions* medication may be directed more immediately towards the affected organ: thus, in illusions of the organ of hearing, applications may be made to the mastoid process; and in those of vision, the ext. belladonna may be given.

*Sedatives.*—Opium, M. Pinel thinks, produces rather than allays excitement. The Commissioners in Lunacy found a difference of opinion to prevail among the medical directors of the various asylums as to the value of this drug; but, upon the whole, they consider the practice of giving it is considerably gaining ground. Dr. Conolly has found it of utility; sometimes giving it only at night, and at others in repeated doses in the day. Grain doses of the acetate of morphia at night, and smaller portions in the day, have been given. Upon the whole, he prefers the *hyosciamus*, giving it in two-drachm doses at night. He adds—

> "But it should never be forgotten in a lunatic asylum, when a patient is noisy at night, that a copious draught of cold water is often a better sedative than any medicine. By this means, and by allowing the patient to wash his hands and face, and by a quarter of an hour's quiet conversation with him, and by causing his bed to be re-adjusted, a patient may occasionally be tranquillized, who would otherwise disturb his neighbours for hours. In other cases, the unexpected offer of a little bread and meat, or bread and cheese and beer, is very successful, although it has not been thought expedient very often to resort to it. These trifles are important, not merely because they give the patient a quiet night, but because they also interrupt the habit of being noisy in the dark."

M. Pinel criticises the treatment of insanity recommended by the late Sir W. Ellis, whom he cites as our most recent author, and elevates into a rank as an authority among us we were not aware he possessed. Indeed, he seems to be under serious mistakes as regards English practice in insanity, when he states it is wholly guided by phrenological ideas, and consisting in a multifarious employment of drugs—*la polypharmacie des Anglais.* Mercury, he tells us, carried to salivation, is in constant employment by both British and American practitioners! Notwithstanding these his exaggerated objections, he recognizes much in the active employment of blood-letting recommended by Ellis, (but we think scarcely sanctioned by our best practitioners,) at the commencement of the disease, which renders it very superior to the expectant plan prevailing in his own country, as tending to prevent the relapses which are often so fatal, even when the original attack may be recovered from.

MORAL AND HYGIENIC TREATMENT.—Passing by the excellent general observations of M. Pinel upon this subject, for which we have not space, we may advert to some of the particulars.

*Separation.*—The separation of the lunatic from all his habitual associations, his friends, relatives, servants, &c., is a most essential preliminary, and the difficulty which attends the attempt to cure the rich in their own abode is well known. By this isolation the invalid is removed from the influence of some of the primary causes which have affected him: and he is spared the sight, always a painful one to him, of those who were first witnesses of his infirmity. This too is the only mode of ensuring his quiet obedience to the directions of his physician. The novelty which surrounds him is a salutary diversion, operating usefully by exciting that spirit of curiosity so conspicuous in the insane. Persons not practically acquainted with lunatics have feared this separation from all their friends would prove a cause of grief, aggravating their condition. There is, in fact, no better mark of insanity than the loss, or perversion, of the natural feelings. So too, it has been thought that the sight of other insane persons would act prejudicially upon them. This is a mere chimera. The feeling of astonishment which usually seizes the lunatic when first brought amid the insane usually proves beneficial, and he at once sees that he shall be obliged to become obedient. He looks up to his physician as his guide and support, soon becomes familiar with the attendants, but very rarely with the other patients. This repugnance which the insane feel towards each other wears off as convalescence approaches. M. Pinel states that the French law is sufficiently stringent to render it impossible for sane persons to become immured. In our own country, however, the Commissioners, during their recent tour, have liberated persons who ought never to have been confined.

*Cerebral Education.*—By this term M. Pinel designates the employment of the various means for combatting the perverted condition of the faculties of the insane. To carry it out requires, on the part of the physician, great elevation of mind, infinite patience, and great powers of observation. All his subordinates must understand the objects he has in view, aid him in carrying them out, and supply his place when absent—so that a continuity of action may be maintained, which will effect much more than he is able to accomplish himself during his short visits.

"A general error prevails, that a lunatic must not be contradicted, and that we should give way to his delusions for fear of exasperating him. This fatal compliance is, of all things, likely to confirm the insanity, and that it should not be indulged in is a principal reason for removing him from his home. We must then oppose ourselves to his habitual delusions, but it requires much tact to do so with effect, and to know when to suspend our opposition.

"An infallible means of education for convalescents is to bring them much together, both during the period of their working hours, and that of their meals and recreations. The intimacies, emulation, and distraction thus procured are of great use, and the absence of such renders the treatment of rich patients so much more difficult. During convalescence, too, the properly regulated visits of friends, when not commenced too soon, are of great utility in restoring the mind to its natural affections and aptitudes. It must never be forgotten, however, that the brain, like any other diseased organ, can only be permanently restored by degrees, and that any precipitancy in employing it upon the exercise of its faculties will retard the cure, or cause relapse. In certain forms of partial, chronic, and calm insanity, the moral treatment may be carried to a more daring extent than is ordinarily justifiable. This is the case, however, only with visionaries, monomaniacs, and melancholics, in whom no organic lesion can be detected. Such persons must not be left too much at rest, and the infliction of pain may be required to arouse the mind from lethargy, or to subdue a dominant idea. The *douche* is the fittest instrument by which this can be effected; but this is only one out of others, the general system being that the life of the patient shall be rendered agreeable only in proportion as he consents to follow the councils of the physician. At the same time, he must be encouraged to occupy his mind in any way likely to break through the chain of dominant ideas."

This is very questionable practice; and the application of force as a means of conquering delusions is, we believe, entirely repudiated in this country, and certainly the principle, if carried out might easily lead to the grossest abuses.

M. Pinel observes, that moral treatment may, in large establishments, be successful in apparently hopeless cases. In these, there are always a large number of patients reputed incurable, who are only so because attempts at their amelioration have never been put into force; and neglected by their friends for numbers of years, they have sank into a state of listless contentment with a situation in which their physical wants are supplied, and they realize a portion of imaginary happiness. Many continue mad merely because they remain among the insane, and from habit, idleness, and fear of having to labour, are content to remain where they are. Such patients may be advantageously submitted to moral treatment after years of confinement, and may require to be subjected to the rudest tests, to oblige them to resume their reasoning powers.

"It is often sufficient to thrust them out of the hospital in spite of themselves. Being at the Salpêtrière, I was struck with the repugnance which a great number of these persons manifested at recovering their liberty, and re-entering the world. Convinced that indolence was the chief cause of this, I proposed to M. Pariset and the administrator to make an extensive experiment. I pointed out to them seventy-two patients, chosen from among those who had been for years reputed incurable; and they were all turned out. Of these, three only returned—the rest finding a means of livelihood." 540.

It is probable that many of these persons had never been mad at all, but sought refuge in this establishment as our poor do in a workhouse.

*Arrangements of an Asylum.*—The author, referring to his larger work upon the subject, offers some observations upon the most appropriate construction of an asylum. These we can only allude to as useful for consideration in conjunction with the numerous suggestions offered by the Commissioners in Lunacy in their Report, derived from the contemplation of the defective state of existing structures in this country. This is a highly important subject just now, when the necessity for increased accommodation for the insane is about to be practically acknowledged. The Commissioners pronounce numbers of existing buildings as positively unfitted for their purposes, and few, if any, are without serious positive defects as regards airing-grounds, sleeping-rooms, &c., or some other important particular. They insist upon some arrangement being entered into for the due classification of patients, which is neglected even in the best-conducted asylums, to the great sacrifice of the comfort of many of the patients, and the retardation of the cure of others. Dr. Pinel proposes the distribution of patients into six classes:—1. Convalescents. 2. Quiet lunatics under treatment. 3. Incurable quiet lunatics and imbeciles. 4. Incurable unquiet lunatics, and epileptics. 5. Furious lunatics in cells;—and 6. The infirmary for accidental diseases and paralytics. Improved arrangements are also required for the admission and discharge of lunatics. It is well known that insanity is curable in a large proportion of cases, when treatment is commenced early, which, if its application had been delayed, would have resisted all measures. "The probability of effecting a cure in the case of a patient who is admitted within one month, is," says the Report on Bethlem, "just double that of one who has been admitted within two months of the commencement of the complaint." Mr. Tuke states, that of 63 cases admitted during the first three months 51 recovered: of 65 between the 3d and 12th month 28; and of 101 after the first year but 31. It is then a lamentable fact, that the vast majority of pauper lunatics are detained in workhouses, possessed of no means of treating them, until the period of cure has passed away: but once sent to the asylums, and even when found incurable, they are allowed to remain there for years, so that these establishments, instead of being places for the cure of insanity, become expensive receptacles of incurable lunatics. The Commissioners state that, out of 984 patients in Hanwell at their visit, only *thirty* were accounted curable! This is indeed a crying grievance. The poor lunatic, often on account of the parsimony of his parish, is only sent at an advanced stage of his disease, when cure is hopeless, or, as often happens, when death is imminent; while the rate-payers are obliged to pay for the prolonged maintenance of old, incurable cases which might be as well provided for in far less expensive establishments. Except under peculiar circumstances, the asylums should be places for early resort and limited residence.

The Commissioners forcibly object to the large *size* of some of the asylums, as Hanwell, rendering the proper disposition of the departments and the due governance of the institution difficult, and the duties of the officers onerous. They consider from 200 to 250 beds as the most convenient for all purposes. We think their objection a valid one.

It is a matter for congratulation that these institutions are about to be converted into *clinical schools* for the observation of the diseases of the mind. It seems almost incredible that the rich field for study which the metropolis supplies should have been so little cultivated, that, with the exception of Sir Alexander Morison, no one, that we know of, has ever delivered a course of lectures upon the subject until the recent ones of Drs. Conolly and Sutherland. Sir A. Morison's lectures were commenced in 1823 and continue to the present time; and the fact of only 150 gentlemen having attended them during this long period proves how little alive medical men have been to the importance of the subject. The consequence is that the treatment of the insane has been, until quite recently, converted into a speciality, enveloped in mystery, conducted with unnecessary harshness and privations, and frequently placed in the hands of persons whose interests ran counter to those of their patients.

All authorities agree in the importance of the office of the *medical superintendent*. His must be an undivided, an omnipotent authority, to which all subsidiary arrangements must be subservient, so that the plans he adopts may be harmoniously carried out in every circumstance that regards the lunatic. The obstacle a very large establishment offers to this, and to the patient being brought sufficiently often in contact with him, is obvious. "He shuts himself up amidst his patients," says M. Pinel, "and consecrates almost every instant of his life to their service, the better to observe, and by observing, to learn to relieve their infirmities. Such an abnegation can only result from the possession of a most elevated mind, and requires the possession of a particular organization, as well as a commanding presence." Whatever qualities the physician may possess, however, single-handed he can do but little, and it is one of the best features of the modern humane system of treatment that *female co-operation* is so much appreciated. The Reports before us contain well-deserved encomia upon the valuable services rendered by the matrons of the various establishments they relate to. Now the revolting appliances formerly in vogue are rapidly disappearing, the kindly nature, ready tact, and fertility of invention so eminently possessed by woman will accomplish much which they failed to achieve. A chief difficulty in carrying out improvements in the treatment of the insane has been found in the deficiency of intelligent and humane *attendants* (olim keepers). This the more so, since the diminution of the employment of mechanical restraint has rendered a greater proportionate number necessary. A superior class of persons can only be induced to undertake so laborious, anxious, and repulsive a situation by the prospect of good remuneration and kind treatment; and, when we consider how much the well-being of an asylum depends upon their exertions, we must allow that their remuneration can scarcely be too high. However well disposed, persons in this rank of life do not always possess the aptitude and requisite information, and we think Sir Alexander Morison's plan of delivering a course of lectures to them upon their duties worthy of imitation. M. Pinel states that we have normal schools for this purpose in England which are very successful. Of their locality we are ignorant. A well-regulated asylum is the best of normal schools.

An important appendix to a lunatic asylum is a *convalescent* establishment. The lunatic who is cured frequently has no means of re-entering society if he is abruptly discharged from the asylum. Some establishments allow the patients to return to meals and at night for a while, and others furnish them with money, as the Adelaide fund at Hanwell. M. Pinel speaks highly of the excellent results which have followed the establishment of a large farm for the temporary reception and employment of those discharged from the Bicêtre.

*Restraint.*—Upon the extent to which employment of this should be carried M. Pinel is entirely silent, which is surprising, considering the position his father assumed upon the question, and the great attention the subject has excited during the last five years. The *practicability* of totally abolishing all mechanical restraint in asylums chiefly destined for the reception of chronic cases of insanity, has been completely demonstrated at Lincoln, Hanwell, and Lancaster. That, however, whether as regards the lunatic himself, his attendants, or his fellow-patients, its indiscriminate proscription, especially in establishments where only recent, and often violent, cases are received, would be most injudicious, we have often stated in this Journal: and we are glad to find that the dispassionate inquiries of the Commissioners have led them to the adoption of a similar view. They state that the opinion is unanimous upon the part of the superintendents of insane establishments that the condition of the insane has improved in proportion as mechanical restraint has been disused. In point of fact, it is nearly practically abolished in public receptacles. In 17 of these, containing 2868 patients when visited there were but 24 persons under moderate restraint; and while the weekly average at Bethlem was 11 in 1839, it was only 3 in 1843. But few medical officers, however, approved of the power of using it being totally withdrawn, and the Commissioners relate several examples of serious accidents resulting from its total disuse. Upon the score of humanity, Mr. Tuke, and numerous other well-qualified observers, prefer its mild employment, as subduing impetuosity with least suffering to the patient. The Commissioners upon this point observe:—

"In those cases where the patient is overpowered by a number of keepers holding his hands during a paroxysm of violence it is said there is no mechanical restraint. Here restraint of some sort or other is manifest; and even in those cases where the patient is forced into a cell by manual strength, and prevented leaving it until his fit of excitement shall have passed, it is difficult to understand also how this can be reconciled with the profession of abstaining from all restraint whatever, so as to be correctly termed 'non-restraint.' It seems to us that these means are only particular modes of restraint, the relative advantages of which must altogether depend upon the results."

They likewise offer a proper caution respecting the *Seclusion* of the patient, which is the substitute for restraint in case of violence.

"As a temporary remedy for very short periods, in cases of paroxysms of high excitement, we believe seclusion is a very valuable remedy. We are convinced, however, that it ought to be used only for very short periods, and that it should not be permitted as a means of managing and treating those persons who are permanently violent and dangerous."

The universal abandonment of restraint would be attended with great expence in employing additional attendants without corresponding advantage, but the contrary, and in private establishments would be impracticable. Its abuse must be guarded against by its infliction requiring the sanction of the physician, and its duration being faithfully recorded. The same precautions are required when seclusion in dark cells is employed.

*Employments and Amusements.*—The importance of these can scarcely be exaggerated, and it is chiefly to their greater development that the improved condition of the insane is attributable. The Commissioners regret that so very large a proportion of lunatic establishments possess insufficient (and several none whatever) accommodation for out of door exercise and employment. Even did not these measures tend, as they most markedly do, to augment the chances of recovery, they would still be invaluable in the eye of the philanthropist, as conferring a share of modified happiness upon beings heretofore deemed incapable of all but mere sensual enjoyments. Pinel fully confirms the statement of observers among ourselves, especially as regards the value of manual labour. Unfortunately there are many patients whose interest can scarcely be excited by any means, owing in great part to their ignorant condition prior to the occurrence of the attack. To these, and others, much benefit has accrued at Hanwell by the endeavour to instruct them in reading and writing; and, although the experiment has only been tried on a small scale, it has been attended with marked success, which is the more gratifying as the results may be of great utility to the individuals after a cure has been effected. Music, it seems to us, has been insufficiently resorted to in this country.

*Religious Services.*—These, when it is considered how often perverted religious ideas give rise to insanity, would seem, *à priori*, inappropriate in a lunatic establishment. If all were admitted indiscriminately to partake in them such would be the case; but where due care has been made in selecting the individuals, and due discretion that the service shall not be too prolonged or too exciting, unmixed good has arisen, according to the testimony of all where the experiment has been tried, a degree of decorum being maintained; incredible to those who had not witnessed it. "The effect," say the Commissioners, "is tranquillizing, and productive of good order and decorum, in a remarkable degree, and in some instances permanently beneficial." M. Pinel chiefly employs this means in convalescence. "Among these unfortunate beings there are many who regain with their reason the desire for those religious observances they have been accustomed to, and who derive from their practice hope and resignation." The Commissioners observe, however, that religious books often form too large a proportion in the libraries provided for the insane. This, and the propriety of admitting particular individuals to join in the services, should be under the complete control of the medical superintendent.

*Diet.*—The lunatic has frequently an excellent and sometimes a voracious appetite. All modern observers agree that his diet should consist of a larger proportion of solid and nourishing food than was formerly deemed fitting. The dietaries of most establishments have been augmented with advantage to the well-being of the patients. As in all large assemblages of poor persons, those of pauper asylums are not sufficiently varied in the description of aliments employed, while the omission of tea and sugar must often be felt as a severe privation. Bethlem Hospital has recently gone to great expense to furnish these luxuries (?) to the inmates of that noble establishment, many of whom belong to a rank of life above that of the pauper, but yet are too needy to bear the expense of purchasing them.

